# The Relation between ACE Gene Polymorphism and the Severity of COVID-19 Infection

**DOI:** 10.1155/2023/4540287

**Published:** 2023-01-04

**Authors:** Yara El-Sayed Marei, Ahmed Abdallah Bayoumy, Hassnaa Mohamed Abulazm Nassar, Bassam Mansour, Asmaa Bakeir Hamady

**Affiliations:** ^1^Medical Microbiology and Immunology Department, Faculty of Medicine, Suez Canal University, Ismailia, Egypt; ^2^Chest Unit Internal Medicine Department, Faculty of Medicine, Suez Canal University, Ismailia, Egypt; ^3^Clinical Pathology Department, Faculty of Medicine, Suez Canal University, Ismailia, Egypt; ^4^Infection and Endemic Disease Department, Faculty of Medicine, Suez Canal University, Ismailia, Egypt

## Abstract

**Introduction:**

The coronavirus disease 2019 (COVID-19) pandemic, which emerged in China at the end of 2019, rapidly spread worldwide. The angiotensin-converting enzyme (ACE) gene contains an insertion/deletion (I/D) polymorphism that leads to a higher serum ACE level which is associated with several diseases and also with a high mortality rate in SARS. Therefore, this study aimed at assessing the association between ACE gene polymorphism and the risk and severity of COVID-19 disease in patients. *Methodology*. Forty-five SARS-CoV-2 infected patients and another random control group of 45 healthy individuals were included. The detection of ACE I/D gene polymorphism in both groups was done by PCR.

**Results:**

53% of infected patients with SARS-CoV-2 had an ACE deletion/deletion genotype (D/D), 27% had an ACE deletion/insertion genotype (D/I), and 20% had an ACE insertion/insertion genotype (I/I). On the one hand, the D/D variant was significantly detected in the COVID-19 patients compared to the control subjects, whereas the I/I variant was significantly detected in the control subjects compared to the COVID-19 patients (*p* = 0.004). The D/D variant subgroup showed the lowest lymphocytic count compared to the D/I or I/I subgroups. In addition, the C-reactive protein was significantly higher and the oxygen saturation was significantly lower in patients with the D/D allele compared to the other subgroups.

**Conclusions:**

ACE gene polymorphism, particularly the DD genotype, was observed to affect the severity of COVID-19 infection.

## 1. Introduction

Coronaviruses (CoV) have emerged as the most common pathogens in respiratory illness outbreaks. They are a broad group of single-stranded RNA viruses that can be found in a variety of animals [1]. Coronaviruses are classified into four genera based on the target host; mammals are infected by *α* and *β*-CoV, whereas birds are affected by *γ* and *δ*-CoV [2].

The world is dealing with the coronavirus disease 2019 (COVID-19) pandemic, which is caused by the severe acute respiratory syndrome coronavirus 2 (SARS-CoV-2) that belongs to *β*-CoV genus of coronaviruses. This pandemic began in China at the end of 2019 and quickly spread throughout the world [3]. In Egypt, the SARS-CoV-2 index case was registered by health authorities on February 14, 2020 [4].

Angiotensin-converting enzyme (ACE) is a component of the renin-angiotensin-aldosterone system (RAAS) that is involved in blood pressure homeostasis. ACE converts angiotensin I (Ang I) to angiotensin II (Ang II) which, through its interaction with the angiotensin II type 1 receptor (AT1R), induces a strong vasoconstriction and triggers proinflammatory, proapoptotic, and profibrotic pathways in the lung and other organs [5].

Another homolog of ACE is ACE2, which is encoded by the human X-chromosome. ACE2 is a typical zinc-metalloproteinase type I transmembrane protein that is expressed ubiquitously in different organs of the human body, such as the heart, lungs, kidneys, intestines, and endothelium [6]. In RAAS, ACE2 contributes to the inactivation of Ang II by hydrolyzing it to Ang-1–7, which stimulates vasodilation and promotes anti-inflammatory, antifibrotic, and antithrombotic actions via the Ang-1–7/Mas receptor axis. Therefore, ACE2 physiologically counters Ang II/AT1R effects [5].

Another major role of ACE2 was identified by the emergence of severe acute respiratory syndrome (SARS) in 2002-2003, caused by SARS-CoV. Human ACE2 acts as a functional receptor that binds to the SARS-CoV spike (S) protein with high affinity, which is considered a critical step in the virus's entry into human cells [7].

The ACE insertion (I)/deletion (D) gene polymorphisms are one of the most frequently defined human polymorphisms. D and I polymorphisms in the ACE gene may result in differences in ACE levels. For instance, the ACE D allele causes an increase in ACE1 level and a decrease in ACE2 level, causing an increased level of angiotensin-2 which may predispose the individual to a variety of disorders including obesity, hypertension, increased cardiovascular risk, and thrombophilia [8]. Furthermore, the ACE D/D gene polymorphism causes the progression of pulmonary edema through increased microvascular permeability, which further worsens the clinical course and prognosis of the acute respiratory distress syndrome (ARDS) and has been linked to a high mortality rate in SARS patients [9].

The lack of any functional ACE gene polymorphisms being linked to SARS infection might be due to a lack of previous studies. Therefore, this study aimed at assessing the association between ACE gene polymorphism and the risk and severity of COVID-19 disease in patients.

## 2. Methodology

A case-control prospective study of the relation between ACE gene polymorphism and the severity of COVID-19 infection was planned. A sample of patients who tested positive for SARS-CoV-2 were compared with a sample of controls who tested negative for SARS-CoV-2, assuming an equal number of cases and controls (*r* = 1). Previous studies have shown that around 73% of positive SARS-CoV-2 patients had the DD allele (*p*1 = 0.73) [10]. For achieving a 90% power (1 − *β* = 0.9) at the 5% level of significance (*α* = 0.05), the sample size to detect a proportion of negative tested subjects of SARS COV2 (*p*2 = 0.304) [11] is 45 cases and 45 controls.

Patients who presented with the COVID-19 characteristic symptoms of fever, cough, and dyspnoea and were admitted to the hospital with an already proven SARS-CoV-2 infection were originally enrolled. Additionally, we looked at 45 healthy subjects who were negative for SARS-CoV-2 using reverse transcription real-time PCR testing. The following were the exclusion criteria: (1) age over 70; (2) chronic kidney disease (CKD) managed by dialysis; and (3) patients with restrictive or obstructive lung diseases.

All age groups were included. Informed consent was taken from each patient to use their data in the current research work. We obtained the approval of the Ethics Committee of the Faculty of Medicine, Suez Canal University (Ethics approval number: Research 4555#).

The following data were collected from each patient in both groups: age, gender, and comorbid conditions (diabetes, cardiovascular diseases, history of active malignancy). In addition, initial peripheral oxygen saturation by pulse oximetry and laboratory tests for complete blood count, C-reactive protein (CRP), and D-dimers were recorded in the COVID-19 group.

A computed tomography of the chest was performed and the extent of the lesions was graded according to the COVID-19 Reporting and Data System (CO-RADS) score (Table 1) [12].

### 2.1. Collection of Specimens

Peripheral blood samples were collected from each patient and control subject in sterile tubes containing EDTA. The blood samples were immediately centrifuged at 1500*g* and stored at −20°C until further processing.

### 2.2. Processing of Specimens

#### 2.2.1. Genomic DNA Extraction

According to the manufacturer's instructions, genomic DNA was extracted from peripheral blood using a commercially available spin-column technique kit for DNA extraction (QIAamp®DNA Blood Mini Kit) (QIAGEN, inc., Hilden, Germany).

The DNA yield and purity were assessed by a spectrophotometer at 260 nm (Nanodrop) (Thermo Fisher Scientific Inc. USA). The ratio of absorbance at 260 and 280 nm (A260/A280) was used to determine the purity of DNA.

#### 2.2.2. Detection of ACE Insertion/Deletion (I/D) Polymorphism by PCR

According to Tiret et al., the ACE insertion/deletion (I/D) polymorphism was amplified by PCR using a specific set of primers (Table 2) [13].

The PCR reaction was done at a final volume of 25 *μ*l containing 1 *μ*L (20 ng) of extracted DNA as a template, 0.5 *μ*l forward primer, 0.5 *μ*l reverse primer (10 Pmol), 12.5 *μ*l of 2 × Master Mix (including 1.5 × PCR buffer, 0.5 mmol/L of dNTPs, 4 mmol/L of MgCl_2_, and 0.08 IU of Taq DNA polymerase), and 10.5 *μ*l double-distilled water.

**Figure 1:**
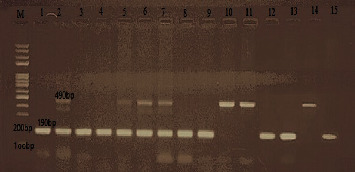
PCR on blood samples of SARS-CoV-2 patients for detection of ACE I/D polymorphism. The I allele is demonstrated as a 490 bp band and the D allele as a 190 bp band.

**Figure 2:**
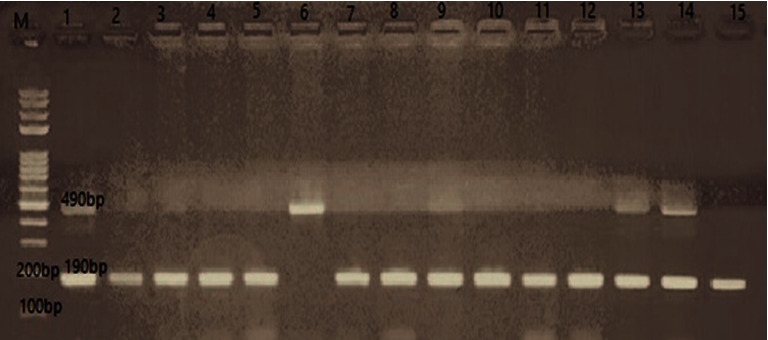
PCR on blood samples of non-SARS-CoV-2 individuals for detection of ACE I/D polymorphism. The I allele is demonstrated as a 490 bp band and the D allele as a 190 bp band.

The DNA was amplified in the thermal cycler (Eppendorf Co., Germany) using the following protocol: initial denaturation (94°C for 2 minutes), followed by 35 cycles of denaturation at 94°C for 1 min, annealing (58°C for 1 minute), and extension (72°C for 2 minutes), with a single final extension of 10 minutes at 72°C. The amplified products were visualized by electrophoresis on 2% agarose gels stained with ethidium bromide and then visualized under ultraviolet (UV) illumination. The amplicons were compared to a molecular-weight DNA ladder with sizes ranging from 100 to 1000 bp (Fermentas, Germany) to visualize three patterns: I/I (490 bp fragment), D/D (190 bp fragment), and I/D (both 490 bp and 190 bp fragments), as depicted in Figures 1 and 2.

### 2.3. Statistical Analysis

All statistical analyses were performed using SPSS version 22.0 (IBM, Armonk, New York, United States). Significance was observed with *p* value < 0.05.

Variables were tested for pattern of distribution using the Kolmogorov–Smirnov test and visual assessment of histograms. Data with normal distribution were expressed as mean ± standard deviation while non-normally distributed variables were presented as median and interquartile range (IQR). To assess possible differences between groups for parametric and nonparametric variables, we used unpaired student *t*, one-way ANOVA (Bonferroni test for in-between group differences), and Mann–Whitney U, Kruskal–Wallis tests, respectively. To compare categorical variables, chi-squared test was used.

For detection of the prediction model of the DD allelic variation, we calculated the odds ratio (OR) and 95% confidence interval (CI) by multivariable logistic regression.

## 3. Results

The results of this study showed that the females were represented almost similarly in both groups with no significant difference (*p* > 0.05). In addition, diabetes mellitus, cardiovascular diseases, and history of active malignancy were similar in both groups (*p* > 0.05). Analysis of the markers of severity related to the SARS-CoV-2, the COVID-19 group revealed low means lymphocytic count, high CRP, and D-dimer tests. Additionally, the COVID-19 patients showed reduced mean oxygen saturation (Table 3).

Regarding the results of conventional PCR for testing the presence of ACE I/D gene polymorphism on peripheral blood samples of patients in both groups as shown in Figures 1 and 2, it was found that 53% of infected patients with SARS-CoV-2 had ACE D/D polymorphism, 27% had ACE D/I polymorphism, and 20% had ACE I/I polymorphism. The control group showed that PCR of blood samples revealed that 29% had ACE D/D polymorphism, 18% had ACE D/I polymorphism, and 53% had ACE I/I polymorphism.

**Figure 3:**
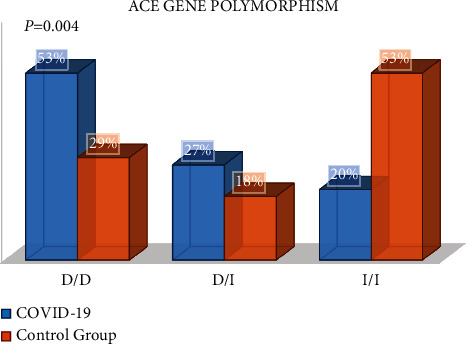
The comparison between the SARS-CoV-2 group and the control group regarding the results of ACE I/D polymorphism by PCR.

The comparison between the SARS-CoV-2 group and the control group regarding the ACE gene allelic variants revealed that the D/D variant was significantly detected in the COVID-19 patients compared to the control subjects (53% and 29%, respectively), whereas the I/I variant was significantly detected in the control subjects compared to the COVID-19 patients (53% and 20%, respectively) (*p* = 0.004), as shown in Figure 3.

The COVID-19 patients with different ACE gene allelic variants showed that age and D-dimer were not different between all variants' subgroups. Regarding the markers of high inflammatory reaction, the D/D variant subgroup showed the lowest lymphocytic count compared to the D/I or I/I subgroups. In addition, the C-reactive protein was significantly higher in the D/D patients compared to the other subgroups. Moreover, oxygen saturation was significantly lower in the D/D subgroups in comparison with other subgroups (Table 4).

Multivariable logistic regression analysis was performed for the detection of the predictors of the DD allelic variation and revealed that no variable in the model appeared to have a significant association with allelic variation (Table 5).

## 4. Discussion

The RAAS is mainly responsible for regulating blood pressure, fluid volume, and electrolyte balance. The physiological homeostasis of the RAAS is regulated by the balance of ACE and ACE2. ACE converts Ang I to Ang II which exerts vasoconstrictive, hypoxic, oxidative, hypertrophic, fibrotic, and inflammatory actions and is involved in the development of various pathologies. ACE2 is responsible for counteracting the adverse effects of angiotensin II through its proteolytic product, angiotensin (1–7) which activates signalling pathways that lead to vasodilation and inhibition of cell proliferation, but also accounts for strong anti-inflammatory, antifibrosing, and antioxidative effects. Therefore, the quantities of the two enzymes have been considered to be the main endogenous regulators of the RAAS [14].

Several observations support the evident association between RAAS imbalance and COVID-19 disease. This is because ACE2 is the main cellular receptor for the SARS-CoV-2, and this interaction results in exhaustion and reduced expression of ACE2, resulting in elevated levels of angiotensin II which could explain the deleterious lung injury observed in SARS-CoV-2 infection [15].

This study was conducted to demonstrate the association between ACE gene polymorphism and the risk and severity of COVID-19 infection. It was found that 53% of infected patients with SARS-CoV-2 had ACE D/D polymorphism, 27% had ACE D/I polymorphism, and 20% had ACE I/I polymorphism. On the one hand, the D/D variant was significantly detected in the COVID-19 patients compared to the control subjects (53% and 29%, respectively), whereas the I/I variant was significantly detected in the control subjects compared to the COVID-19 patients (53% and 20%, respectively) (*p* = 0.004).

There is an evidence that the ACE I/D polymorphism influences plasma ACE concentration, with D/D genotype individuals having approximately doubled plasma ACE concentrations (as the D allele of the ACE I/D gene polymorphism mediates higher ACE expression) compared to I/I genotype individuals, and the I/D genotype individuals having intermediate concentrations [16].

The I/D polymorphism in the ACE gene has been previously examined in relation to variable diseases' susceptibility and consequences, with contrasting results. According to a meta-analysis, ACE gene polymorphisms, particularly the D/D genotype, may increase the risk of respiratory disease with pulmonary hypertension [17]. Some researchers have found a link between the prevalence of the ACE gene polymorphism and the progression of SARS [18].

In Italy, Annunziata et al. found that 73% of critically ill COVID-19 patients had the D/D polymorphism, 23% had the I/D polymorphism, and just 8% had the I/I polymorphism [10]. Moreover, Amar and colleagues in Pakistan found a probable correlation between the ACE I/D gene polymorphism and COVID-19 prevalence, fatalities, and recovery rate in a meta-regression analysis. They discovered that nations with a higher frequency of the D allele had higher COVID-19 infection prevalence and fatalities, especially when compared to European and Asian countries [19]. In addition, Mir et al. in Saudi Arabia reported that the frequency of the D allele was found to be significantly higher among COVID-19 patients than in the healthy controls [20].

Regarding the markers of high inflammatory reaction, our study revealed that the D/D variant subgroup showed the lowest lymphocytic count compared to the D/I or I/I subgroups. In addition, the C-reactive protein was significantly higher in the D/D patients compared to the other subgroups. Moreover, oxygen saturation was significantly lower in the D/D subgroups in comparison with other subgroups.

Similar results were reported by Gómez et al. in Spain, who conducted research on 204 Spanish SARS-CoV-2-infected patients and found a hypertension-dependent link between the ACE-DD genotype and severe COVID-19 infection [21]. Furthermore, Verma et al. and colleagues in India found that the ACE1 DD genotype and the frequency of the D allele were significantly higher in severe COVID-19 patients [22]. Similarly, Mir et al. in Saudi Arabia showed that the ACE-DD genotype was strongly associated with increased COVID-19 severity (*p* < 0.013) and mortality (*p* < 0.008) [20].

In contrary to our results, Çelik et al. in Turkey investigated the relationship between ACE gene I/D polymorphism, ACE2 receptor gene rs2106809, and rs2285666 polymorphism, and COVID-19 severity in 155 COVID-19 patients who were divided into three groups (mild, moderate, and severe) according to clinical symptoms. They concluded that ACE gene I/D, ACE2 receptor gene rs2106809, and rs2285666 polymorphisms were not associated with the severity of COVID-19 infection [23]. Also, Möhlendick et al. in Germany found that the ACE gene polymorphism was not related to infection risk or severity of COVID-19 [11].

Functionally, COVID-19 patients with the D/D genotype may have an unopposed abundance of angiotension II protein in their blood as a result of the following: increased D allele-mediated excessive ACE expression on one hand and downregulation of ACE2 receptor because of SARS-CoV-2 engagement on the other, thus triggering downstream deleterious effects, the most notable of which is acute lung injury [15].

This RAAS imbalance caused by ACE abundance could possibly explain why COVID-19 infection complications are more severe with the D/D genotype.

In our study, multivariable logistic regression analysis was performed for the detection of the predictors of the DD allelic variation and revealed that no variable in the model appeared to have a significant association with allelic variation (Table 5). Gómez et al. in Spain conducted a multiple logistic regression analysis and showed that hypertension (*p* = 0.02; OR = 2.26, 95% CI = 1.12–4.63) and male gender (*p* = 0.002; OR = 3.15, 95% CI = 1.56–6.66) remained as independent significant predictors of COVID-19 severity [21]. Also, Delanghe et al. concluded that the ACE1 D/I polymorphism independently contributes (*p* = 0.0076) to COVID-19 mortality in a multivariate regression mode [24].

Mir et al. performed multivariate logistic regression analysis to estimate the association between ACE-I/D genotypes and risk to COVID-19. The results indicated that in the codominant model, the ACE-ID genotype was strongly associated with increased COVID-19 severity, with OR 2.20 (95%) CI = (1.08–4.46), RR = 1.34 (1.04–1.72), *p* < 0.020. Similarly, ACE-DD genotype was strongly associated with increased COVID-19 severity with OR 2.37, (95%) CI = (1.19–4.70), RR = 1.39 (1.09–1.77), *p* < 0.013. They concluded a potential dominant effect of ACE-DD genotype and D allele on COVID-19 severity in their study group [20].

The study has certain limitations including the relatively smaller sample size. This is attributed to limited access to data obtained from COVID-19 hospitalized patients and difficulty importing kits and chemicals during the COVID-19 era. Nevertheless, more future research with a larger sample size is required to better understand the association of this polymorphism with the severity of COVID-19 infection.

## 5. Conclusions

ACE gene polymorphism, particularly the DD genotype, was observed to affect the severity of COVID-19 infection.

## Figures and Tables

**Table 1:** The findings of CO-RADS score for grading of CT chest [12].

CO-RADS score	CT finding
CO-RADS 1	CT is normal or there are findings indicating a noninfectious disease
CO-RADS 2	The level of suspicion of COVID-19 infection is low, and CT findings are consistent with other infections
CO-RADS 3	The presence of COVID-19 infection is unknown or inconclusive, and CT abnormalities suggest infection, but it is unclear whether COVID-19 is involved or not
CO-RADS 4	The level of suspicion is high, and most CT findings, such as unilateral ground-glass, confluent, or multifocal consolidations without a typical site or any other characteristic findings, are suspicious but not typical
CO-RADS 5	The level of suspicion is high with typical CT findings
CO-RADS 6	RT-PCR positive for SARS-CoV-2

CO-RADS, COVID-19 reporting and data system.

**Table 2:** Primers used to detect the ACE insertion/deletion (I/D) gene polymorphism.

Gene	Sequence (5′ eq3′)	Size (bp)	Reference
ACE	F: 5′-CTGGAGACCACTCCCATCCTTTCT-3′ R: 5′-GATGTGGCCATCACATTCGTCAGAT-3′	I allele: 490 bp D allele: 190 bp	[13]

**Table 3:** The baseline data.

Variable		COVID-19 group, *N* = 45	Control group, *N* = 45	*P* value
Age^a^ (years)		63 (53–68)	61 (54–67)	0.897
Gender (female)^c^		21 (47)	22 (49)	1.00

Comorbidities^c^	(i) DM	16 (36)	12 (27)	0.495
	(ii) Malignancy	3 (7)	5 (11)	0.714
	(iii) Cardiovascular diseases	4 (9)	6 (13)	0.739

Laboratory data	Lymphocytic count^b^ (*μ*l)	1.16 ± 0.49		
	CRP^a^ (mg/L)	37 (24–68)		
	D-dimer^a^ (mg/L)	0.68 (0.38–1.08)		

Oxygen saturation^b^ (%)		81.2 ± 9.4		

^a^Data were expressed as median and IQ. ^b^Data were expressed as mean ± SD. ^c^Data were expressed as *n* and %.

**Table 4:** The analysis of the markers of severity for COVID-19 patients with different ACE gene allelic variants.

	ACE gene polymorphism			*P* value
	D/D	D/I	I/I	
Age^a^ (years)	63 (48–66)	69 (56–71)	66 (46–68)	0.232
Lymphocytic count^b^ (*μ*l)	0.88 ± 0.36^*α*^	1.25 ± 0.38^*β*^	1.8 ± 0.34^*μ*^	**<0.001** ^*∗*^
CRP^a^ (mg/L)	63^*α*^ (36–81)	33^*β*^ (22–42)	24^*β*^ (23–31)	**0.001** ^*∗*^
D-dimer^a^ (mg/L)	0.79 (40–1.18)	0.52 (37–89)	0.78 (29–2.14)	0.417
Oxygen saturation^b^ (%)	77.8 ± 10.2^*α*^	85.5 ± 8.1^*β*^	84.8 ± 5.1^*β*^	**0.028** ^*∗*^

^*∗*^Statistically significant results. ^a^Data were expressed as median and IQ. ^b^Data were expressed mean ± SD. Significant difference between *α* and *β*. Significant difference between *α* and *μ*. Significant difference between *β* and *μ.*

**Table 5:** Multivariable logistic regression for prediction of the DD allelic variation.

Variables in the equation	OR	95%		Sig.
		Upper	Lower	
Age	n/a	n/a	n/a	0.988
Gender (1)	0.875	0.332	2.304	0.995
Diabetes (1)	9.063	2.275	36.387	0.988
Carcinoma (1)	1.400	1.156	1.695	0.990
Cardiac diseases (1)	1.414	1.160	1.721	0.988
O_2_ saturation	n/a	n/a	n/a	0.996
Lymphocyte count	n/a	n/a	n/a	0.989
D-dimer	n/a	n/a	n/a	0.987
CRP	n/a	n/a	n/a	0.983
Constant	n/a	n/a	n/a	0.985

## Data Availability

The datasets generated during and/or analyzed during the current study are available from the corresponding author upon request.
